# Risk Associated with the *LEPR* rs8179183 GG Genotype in a Female Korean Population with Obesity

**DOI:** 10.3390/antiox9060497

**Published:** 2020-06-06

**Authors:** Kyunghye Jang, Gurum Shin, Hye Jin Yoo, Jong Ho Lee, Minjoo Kim

**Affiliations:** 1 National Leading Research Laboratory of Clinical Nutrigenetics/Nutrigenomics, Department of Food and Nutrition, College of Human Ecology, Yonsei University, Seoul 03722, Korea; jkh911127@naver.com (K.J.); gloria1261@naver.com (G.S.); hyejin10432@hanmail.net (H.J.Y.); jhleeb@yonsei.ac.kr (J.H.L.); 2 Research Center for Silver Science, Institute of Symbiotic Life-TECH, Yonsei University, Seoul 03722, Korea; 3 Department of Food and Nutrition, College of Life Science and Nano Technology, Hannam University, Daejeon 34054, Korea

**Keywords:** metabolically healthy obesity, metabolically unhealthy obesity, leptin receptor gene, female, Korean population

## Abstract

The difference between metabolically healthy obesity (MHO) and metabolically unhealthy obesity (MUO) phenotypes might be partly attributable to genetic traits modulating body fat distribution and other obesity-related metabolic traits, specifically with regard to *LEPR* rs8179183 in Korean women with obesity. A total of 177 females with obesity participated in the study and were grouped by genotype (GC or GG) and metabolic health status (MHO and MUO). Between the MHO and MUO groups, significant differences were found in waist circumference, waist-to-hip ratio, lipid profiles, glucose-related markers, biomarkers of liver health, adiponectin, oxidative stress markers, whole fat area (WFA), and subcutaneous fat area (SFA) at the level of the L1 vertebra, and WFA and visceral fat area (VFA) at the level of the L4 vertebra. Lipid profiles, glucose-related markers, adipokines, oxidative stress markers, and WFA and VFA at the L4 level were significantly different between the GC and GG genotypes. Notably, the individuals with the MUO phenotype and the GG genotype had the least favorable values of glucose-related markers, lipid profiles, adipokines, oxidative stress markers, and regional fat distribution. These observations suggest that the development of obesity-related metabolic traits is highly associated not only with the rs8179183 genotype but also with metabolic status in Korean females with obesity.

## 1. Introduction

Obesity-related disease complications reduce life quality and expectancy and increase health-care costs. Some studies have suggested that obesity does not always accompany metabolic abnormalities and an increased risk of cardiometabolic complications. Because of the lack of generally accepted criteria to identify metabolically healthy obesity (MHO), its prevalence varies widely among studies. Moreover, the prognostic value of MHO is hotly debated, chiefly because it tends to shift progressively toward metabolically unhealthy obesity (MUO) [1].

Recently, genome-wide association studies have identified a set of loci harboring genes possibly controlling both the distribution of extra body fat and the metabolic profile associated with excess adiposity (i.e., MHO or MUO). To date, ten more genetic variants have been associated with a reduced risk of developing metabolic abnormalities (such as dyslipidemia, hypertension, and type 2 diabetes) and consequent cardiovascular disease (CVD), even in people whose body mass index (BMI) is in the obese range [1]. Paradoxically, a study conducted in a population from the UK Biobank [2] showed that 11 of these genetic variants are more common in individuals with a higher BMI and body fat mass percentage, thus indicating that the genetic score characterizing the MHO phenotype is associated with an increased adiposity range [1]. Several studies have revealed that variant-related changes in the expression of those genes and in the levels or functions of their protein products might lead to variability in fat distribution, thus linking obesity to metabolic abnormalities [3,4].

Indeed, there is genetic evidence that several genes differentially expressed in various fat compartments might be involved in the regulation of fat distribution and obesity-related metabolic traits. However, there are no concise reports on the genotypic effect of the leptin receptor gene (*LEPR*) on fat distribution and obesity-related metabolic abnormalities in a Korean population. Several common polymorphisms of *LEPR* have been found, but only a few appear to have a major effect on obesity [5,6]. For example, in several ethnicities, significant links have been observed between *LEPR* polymorphisms and several variables concerning adiposity and body composition [7,8,9]. Therefore, we hypothesized that the difference between MHO and MUO phenotypes might be partly attributable to genetic traits modulating body fat distribution and other obesity-related metabolic traits, specifically with regard to the *LEPR* rs8179183 polymorphism in Korean female subjects with obesity.

## 2. Materials and Methods

### 2.1. Study Subjects

Study subjects were recruited through advertisements by the Clinical Nutrigenetics/Nutrigenomics Laboratory at Yonsei University from May 2015 to February 2018. Volunteers who agreed to participate were screened to measure BMI and assess all personal histories of disease. After screening, female subjects who were 20–65 years old with obesity (BMI ≥ 25 kg/m^2^) were enrolled. The definition of MUO in the present study was the presence of three or more of the following five criteria: systolic blood pressure (BP) ≥ 130 mmHg or diastolic BP ≥ 85 mmHg, triglycerides ≥ 150 mg/dL, high-density lipoprotein (HDL) cholesterol < 50 mg/dL, fasting glucose ≥ 100 mg/dL, and waist circumference ≥ 85 cm. Subjects who did not fulfill the definition of MUO were considered to have MHO. The exclusion criteria included CVD, cancer, immune disease, hepatic disease, renal disease, pregnancy, and regular dietary supplement use. Additionally, subjects who participated in a weight loss program (within the last two months) or had treatment for obesity were excluded. All participants provided written informed consent, and the Institutional Review Board of Yonsei University approved the study protocol, which complied with the Declaration of Helsinki. The sample size was determined and calculated using the R software v.4.0.0 with the package ”pwr”. In an exploratory pilot study, the waist circumference in the MUO group was 95.8 ± 4.90 cm (mean ± standard deviation), which was higher than that in the MHO group (92.7 ± 5.99 cm). The sample size for this study was determined using a two-sample *t*-test power calculation with an effect size of *d* = 0.539, a power of 0.8, and a level of significance of α = 0.05. The results indicated that a minimum of 35 subjects per group was needed, and thus, we selected an MUO-to-MHO ratio of 1:3.5 to increase statistical power.

### 2.2. Somatometric Measurements

Body weight, height, waist circumference, hip circumference, systolic BP, and diastolic BP were collected for all subjects [10]. Somatometric data on abdominal fat distribution were measured at the levels of the L1 and L4 vertebrae via computed tomography (CT; GE Medical System HiSpeed Advantage system, Milwaukee, WI, USA). Body composition, including fat mass, lean body mass, and fat percentage, was measured via dual-energy X-ray absorptiometry (DEXA; Discovery A, Hologic Inc., Bedford, MA, USA).

### 2.3. Laboratory Assessments

To assess the subjects’ lipid profiles, serum triglyceride, total cholesterol, and HDL cholesterol were measured using kits and an autoanalyzer; low-density lipoprotein (LDL) cholesterol was calculated using the Friedewald formula. Fasting serum glucose, insulin, and the homeostatic model assessment of insulin resistance (HOMA-IR) index were also assessed using the methods described in a previous study [11]. An electrochemiluminescence immunoassay (ECLIA) was employed for the quantitative measurement of C-peptide (Roche, Mannheim, Germany). A Hitachi 7600 autoanalyzer (Hitachi, Tokyo, Japan) was used to measure serum aspartate transaminase (AST), alanine aminotransferase (ALT), and γ-glutamyltranspeptidase (γGTP) levels. Plasma adiponectin concentrations were assessed with an enzyme immunoassay [12], and leptin was evaluated with a human leptin ELISA kit (Millipore, Darmstadt, Germany) and a SpectraMax190 device (Molecular Devices, Shanghai, China). Plasma malondialdehyde (MDA) was measured using a thiobarbituric acid reactive substances (TBARS) assay kit (ZeptoMetrix Co., Buffalo, NY, USA). Plasma oxidized (ox)-LDL was examined using an enzyme immunoassay (Mercodia AB, Uppsala, Sweden). The resulting color reaction was monitored at 450 nm with a Wallac 1420 Victor2 multilabel counter (PerkinElmer Life Sciences, Boston, MA). Urinary 8-epi-prostaglandin F_2α_ (8-epi-PGF_2α_) was measured with a urinary isoprostane ELISA kit (Oxford Biomedical Research Inc., Rochester Hills, MI, USA).

### 2.4. Statistical Analysis

To evaluate differences in biochemical and clinical characteristics according to genotype and metabolic health status, the independent *t*-test, the Mann–Whitney *U*-test, and Kruskal–Wallis ANOVA were performed using IBM SPSS Statistics 25.0 (Chicago, IL, USA). A logarithmic transformation was performed for the skewed variables, and significance is reported based on a two-tailed *p*-value < 0.05.

## 3. Results

### 3.1. Clinical Characteristics by Metabolic Health Status

The female participants with obesity were divided into two groups according to their metabolic health status: MHO (*n* = 138) and MUO (*n* = 39). Significant differences were found in the following: waist circumference; waist-to-hip ratio (WHR); lipid profiles including triglyceride, total cholesterol, HDL cholesterol, and LDL cholesterol; and glucose-related markers including glucose, insulin, HOMA-IR, and C-peptide (Table 1).

With the exception of AST, biomarkers of liver health were significantly increased in the MUO group. Conversely, adiponectin, one of the most abundant and well-studied adipokines, was markedly decreased in the MUO group. Oxidative stress was also profoundly aggravated in this group, as indicated by MDA (*p* = 0.009) and ox-LDL (*p* = 0.001) levels. Through DEXA and CT measurements, we found that whole fat area (WFA) and subcutaneous fat area (SFA) at the L1 level and WFA and visceral fat area (VFA) at the L4 level were significantly increased in the MUO group.

### 3.2. Clinical Characteristics by the LEPR rs8179183 Genotype

Among the 177 Korean females with obesity in this study, 26 carried the GC genotype and 151 carried the GG genotype. Significant genotype effects of *LEPR* rs8179183 on triglyceride, HDL cholesterol, LDL cholesterol, glucose, HOMA-IR, and C-peptide levels were observed (Table 2).

Two of the most abundant and well-studied adipokines, adiponectin and leptin, were significantly decreased and increased, respectively, in individuals with the GG genotype. Moreover, three reliable oxidative stress markers were markedly increased in individuals with the GG genotype compared to those with the GC genotype (all *p* < 0.05). Participants with the GG genotype had larger fat deposits than those with the GC genotype, particularly in terms of WFA and VFA at the L4 level.

### 3.3. Higher Metabolic Risk in MUO Individuals with the GG Genotype

To determine the combined effects of metabolic health status and the *LEPR* rs8179183 genotype, we subdivided the participating Korean women with obesity into four groups, as follows: MHO and the GC genotype (*n* = 23), MHO and the GG genotype (*n* = 115), MUO and the GC genotype (*n* = 3), and MUO and the GG genotype (*n* = 36). The overall differences in biochemical and clinical characteristics among the four groups are presented in Table 3.

Individuals with MHO and the GG genotype showed significant decreases in HDL cholesterol and adiponectin compared to those with the GC genotype, though glucose, HOMA-IR, C-peptide, and MDA increased. Those with MUO and the GG genotype had higher levels of glucose, 8-epi-PGF_2α_, and MDA than those with the GC genotype. Compared to individuals with MHO and the GC genotype, those with MUO and the same genotype had higher WHR, triglyceride, HDL cholesterol, and VFA at the L4 level. Individuals with MUO and the GG genotype had a greater waist circumference and, correspondingly, a higher WHR than individuals with MHO and the same genotype. Additionally, all lipid profiles, all glucose-related markers (except for insulin), MDA, ox-LDL, WFA and SFA at the L1 level, and WFA and VFA at the L4 level were significantly higher in individuals with MUO and GG than in those with MHO and GG, whereas the opposite was true for adiponectin.

## 4. Discussion

The present study identified that the *LEPR* rs8179183 GG genotype has an aggravating effect on several health-related variables in Korean females with obesity, particularly those with an unhealthy metabolic health status. The effect of genotype on subjects with obesity was significant in terms of lipid profiles, glucose-related markers, adipokines, oxidative stress markers, and regional fat areas at the level of the L4 vertebra. In previous studies, several single-nucleotide polymorphisms (SNPs) of *LEPR*, including rs8179183, have been shown to be associated with a variety of chronic diseases [13]. However, few studies have assessed the association between *LEPR* polymorphisms and the risk of CVD, and the results are still controversial. Ruaño et al. [14] found that among Caucasian patients, carriers of the rs8179183 G allele were less likely to gain significant weight than CC carriers when prescribed the atypical antipsychotic drug risperidone. Specifically, in CC carriers, risperidone had its usual effect on weight, but CG and GG carriers had a lower likelihood of gaining weight. Nonetheless, clinical differences and similarities between the CG and GG genotypes were not examined.

Due to the genetic differences among ethnicities, we evaluated differences between the GC and GG genotypes in a Korean population with obesity according to metabolic health status. In a previous Korean population study, *LEPR* rs8179183 was associated with hypercholesterolemia, with the C allele of rs8179183 exhibiting a protective effect against abnormal HDL cholesterol levels [15]. Another Asian study demonstrated that the rs8179183 polymorphism was associated with LDL cholesterol and leptin levels [16]. Wauters et al. [17] also found *LEPR* missense SNPs, including rs8179183, to be associated with insulin in women with obesity and impaired glucose tolerance; furthermore, these SNPs have been linked to insulin-related phenotypes [18]. The results of the present study were similar to those of the studies mentioned above. Thus, our work corroborated prior evidence that *LEPR* is associated with IR, which is a crucial feature of metabolic syndrome (MetS) [19].

In addition to the genetic effect of *LEPR* rs8179183, an individual’s metabolic health status influences several obesity-related metabolic traits. Specifically, MUO individuals with the GG genotype showed the highest waist circumference, lipid profiles (triglyceride, total cholesterol, and LDL cholesterol), glucose and related markers (HOMA-IR and C-peptide), γGTP, adiponectin, oxidative stress markers (8-epi-PGF_2α_, MDA, and ox-LDL), and WFA and VFA at the L4 vertebra, though their HDL cholesterol levels were lower than those of the other three groups. Angel-Chávez et al. [20] reported that the C allele was associated with nonobesity markers, such as healthy BMI, low skinfold thickness, and low girth measurements, in Mexican children and adolescents. Individuals carrying the variant allele C also exhibited a higher frequency of MetS (28.1%) than noncarriers, though the difference was nonsignificant [20]. In a nondiabetic Afro-Caribbean population, individuals carrying the C allele had a higher BMI and waist circumference than those with the GG genotype [21]. Taken together, we speculate that these conflicting results of genetic effects were caused by the various ethnicities, ages, and sexes of the subjects as well as their metabolic health states.

A myriad of studies has demonstrated close associations between obesity and leptin, a well-known adipocyte-derived and anti-obesity hormone. Higher leptin concentrations and decreased sensitivity to leptin can normally be found in people with obesity [22], and this hormone acts through the LEPR, the I-type cytokine receptor. As MetS describes a cluster of metabolic abnormalities, leptin may be a significant factor linking obesity, MetS, and CVD. Waist circumference and female sex emerged as independent predictors of leptin levels, which correlate positively with MetS [23]. Leptin resistance is defined as a lack of response to exogenous leptin and an attenuated response to an increased level of endogenous leptin [24]. Recent studies have proposed that leptin resistance may promote IR and cause the abnormal accumulation of lipids in the liver and cardiac and skeletal muscle, reducing fatty acid oxidation and consequently leading to obesity and MetS [25].

MetS is accompanied by oxidative stress in humans and animals. Hyperleptinemia and leptin resistance may upregulate the generation of reactive oxygen species (ROS), increasing oxidative stress and promoting inflammation [26]. Oxidative stress is not only a consequence of MetS but also a cause and an underlying link in the pathogenesis of MetS. Maslove et al. [27] demonstrated that oxidative stress causes IR in adipocytes and contributes to the increased secretion of leptin, interleukin-6, and tumor necrosis factor-α by adipocytes; additionally, the impact of the resulting ROS leads to the decreased adipocyte secretion of adiponectin. In the present study, the individuals with the GG genotype showed significantly higher leptin and lower adiponectin levels than those with the GC genotype, with markedly increased levels of oxidative stress markers. Taking into consideration that obesity itself can cause oxidative stress [27], we found that the *LEPR* rs8179183 GG genotype specifically affects oxidative stress markers and alters leptin and adiponectin levels.

The limitation of this study was that the MUO group with the GC genotype was very small for comparison. However, proper statistical analyses were performed, and the results are clear. Moreover, this study was only conducted in a Korean population, and future studies are needed to generalize the results with a larger sample size in the entire metabolically affected population.

In conclusion, *LEPR* rs8179183 is associated with metabolic health status in a Korean population with obesity, and this is the first report of MUO individuals with the GG genotype having the most unfavorable values of glucose-related markers, lipid profiles, adipokines, oxidative stress markers, and regional fat distribution. These observations suggest that the development of obesity-related metabolic traits is highly associated with not only the rs8179183 genotype but also metabolic status in Korean females, and these specific factors might have a deleterious impact on the development and progression of chronic diseases.

## Figures and Tables

**Table 1:** Biochemical and clinical characteristics of female subjects with obesity according to metabolic health status.

	MHO (*n* = 138)		MUO (*n* = 39)		*p*
Age (y)	44.1	±9.31	45.5	±6.46	0.392
Weight (kg) ^†^	66.9	±6.09	68.0	±6.42	0.369
BMI (kg/m^2^)	26.6	±1.51	26.9	±1.30	0.266
Waist circumference (cm)	91.3	±5.44	94.9	±4.67	<0.001
WHR	0.90	±0.05	0.93	±0.05	0.001
Systolic BP (mmHg)	115.5	±12.3	120.3	±16.7	0.101
Diastolic BP (mmHg)	71.5	±8.43	74.4	±10.7	0.078
Triglyceride (mg/dL) ^†^	101.1	±39.9	193.0	±58.9	<0.001
Total cholesterol (mg/dL)	197.7	±37.2	216.5	±34.7	0.005
HDL cholesterol (mg/dL) ^†^	57.2	±12.1	44.7	±9.41	<0.001
LDL cholesterol (mg/dL)	124.4	±34.8	137.8	±32.2	0.033
Glucose (mg/dL) ^†^	95.2	±8.01	103.2	±12.1	<0.001
Insulin (μIU/dL) ^†^	11.9	±4.45	13.5	±5.55	0.041
HOMA-IR *^†^*	2.81	±1.17	3.45	±1.48	0.003
C-peptide (ng/mL) ^†^	1.89	±0.64	2.26	±0.68	0.003
AST (IU/L) ^†^	20.3	±5.33	23.3	±9.40	0.063
ALT (IU/L) ^†^	16.9	±7.19	22.4	±13.6	0.001
γGTP (U/L) ^†^	19.5	±15.5	25.3	±14.2	0.001
Adiponectin (ng/mL) ^†^	8.57	±5.95	6.66	±5.11	0.003
Leptin (ng/mL)^†^	21.7	±10.3	23.6	±8.45	0.129
8-epi-PGF_2α_ (pg/mg creatinine) ^†^	1492.2	±496.8	1633.3	±534.7	0.077
MDA (nmol/mL) ^†^	7.53	±2.49	8.60	±1.99	0.009
Ox-LDL (U/L) ^†^	64.3	±20.2	77.4	±21.7	0.001
Measurements from DEXA and CT					
Fat percentage (%) ^†^	33.2	±3.14	33.5	±2.92	0.499
Fat mass (g)	22,802.7	±3340.4	23,366.7	±3157.2	0.347
Lean body mass (g) ^†^	43,624.6	±4014.4	44,209.4	±4327.3	0.450
L1 vertebra					
Whole fat area (cm^2^)	237.7	±59.4	262.1	±41.7	0.005
Visceral fat area (cm^2^)	92.0	±36.6	103.4	±32.6	0.081
Subcutaneous fat area (cm^2^)	145.7	±35.2	158.7	±30.0	0.037
L4 vertebra					
Whole fat area (cm^2^)	319.6	±49.5	343.6	±35.2	0.001
Visceral fat area (cm^2^)	91.4	±27.6	109.7	±2.35	<0.001
Subcutaneous fat area (cm^2^)	228.3	±42.9	233.9	±33.5	0.454

Mean ± S.D. ^†^ tested following logarithmic transformation. *p* values were derived from an independent *t*-test.

**Table 2:** Biochemical and clinical characteristics of female subjects with obesity according to the *LEPR* rs8179183 genotype.

	GC (*n* = 26)		GG (*n* = 151)		*p*
Age (y)	45.7	±10.0	44.2	±8.54	0.241
Weight (kg) ^†^	67.0	±6.26	67.2	±6.16	0.944
BMI (kg/m^2^)	26.6	±1.67	26.7	±1.44	0.485
Waist circumference (cm)	91.0	±5.65	92.3	±1.11	0.376
WHR	0.90	±0.05	0.91	±0.05	0.224
Systolic BP (mmHg)	113.2	±14.0	117.1	±13.4	0.187
Diastolic BP (mmHg)	70.3	±10.0	72.5	±8.82	0.304
Triglyceride (mg/dL) ^†^	98.1	±43.0	125.4	±60.2	0.025
Total cholesterol (mg/dL)	192.2	±40.8	203.5	±36.7	0.179
HDL cholesterol (mg/dL) ^†^	59.6	±12.6	53.5	±12.5	0.014
LDL cholesterol (mg/dL)	113.0	±36.0	129.9	±33.9	0.020
Glucose (mg/dL) ^†^	86.1	±6.90	98.8	±8.77	<0.001
Insulin (μIU/dL) ^†^	10.7	±3.13	12.5	±4.93	0.073
HOMA-IR ^†^	2.27	±0.72	3.07	±1.31	0.001
C-peptide (ng/mL) ^†^	1.63	±0.43	2.03	±0.68	0.004
AST (IU/L) ^†^	20.7	±4.07	21.0	±6.88	0.392
ALT (IU/L) ^†^	18.1	±7.96	18.1	±9.47	0.836
γGTP (U/L) ^†^	21.0	±20.5	20.7	±14.4	0.186
Adiponectin (ng/mL) ^†^	11.1	±9.45	7.64	±4.79	0.027
Leptin (ng/mL) ^†^	18.1	±5.94	22.9	±10.3	0.040
8-epi-PGF_2α_ (pg/mg creatinine) ^†^	1372.1	±520.0	1549.7	±502.2	0.038
MDA (nmol/mL) ^†^	6.75	±1.40	8.04	±2.53	0.002
Ox-LDL (U/L) ^†^	59.2	±18.6	68.6	±21.4	0.040
Measurements from DEXA and CT					
Fat percentage (%) ^†^	33.4	±3.03	33.2	±3.10	0.955
Fat mass (g)	22,888.0	±3357.8	22,933.7	±3301.7	0.931
Lean body mass (g) ^†^	43,426.5	±3970.2	43,809.8	±4108.9	0.712
L1 vertebra					
Whole fat area (cm^2^)	242.2	±60.3	243.2	±56.4	0.865
Visceral fat area (cm^2^)	98.7	±47.7	97.7	±33.7	0.886
Subcutaneous fat area (cm^2^)	143.5	±26.0	149.5	±35.7	0.447
L4 vertebra					
Whole fat area (cm^2^)	304.2	±46.4	328.5	±47.1	0.021
Visceral fat area (cm^2^)	84.5	±30.7	97.3	±27.2	0.035
Subcutaneous fat area (cm^2^)	219.8	±41.6	231.2	±40.8	0.185

Mean ± S.D. ^†^ tested following logarithmic transformation. *p* values were derived from the Mann–Whitney *U*-test.

**Table 3:** Overall differences in biochemical and clinical characteristics among female subjects with obesity according to metabolic status and *LEPR* rs8179183 genotype.

	MHO with GC (*n* = 23)		MHO with GG (*n* = 115)		MUO with GC (*n* = 3)		MUO with GG (*n* = 36)		*p*
Age (y)	45.0	±10.4	44.0	±9.11	50.7	±4.93	45.1	±6.43	0.423
Weight (kg) ^†^	67.3	±6.19	66.9	±6.09	65.0	±7.87	68.2	±6.35	0.671
BMI (kg/m^2^)	26.5	±1.59	26.6	±1.50	26.9	±2.58	26.9	±1.20	0.338
Waist circumference (cm)	90.6	±5.70	91.4	±5.40	94.5	±4.64	95.0	±4.74 *^d^*	0.004
WHR	0.89	±0.04	0.91	±0.05	0.96	±0.02 *^c^*	0.93	±0.05 *^d^*	0.001
Systolic BP (mmHg)	111.5	±13.6	116.3	±12.0	126.0	±12.3	119.8	±17.1	0.109
Diastolic BP (mmHg)	69.1	±9.10	72.0	±8.25	79.3	±14.5	74.0	±10.4	0.227
Triglyceride (mg/dL) ^†^	88.4	±34.3	103.7	±40.6	172.7	±27.3 *^c^*	194.7	±60.7 *^d^*	<0.001
Total cholesterol (mg/dL)	192.3	±42.8	198.8	±36.1	191.3	±24.2	218.6	±34.9 *^d^*	0.015
HDL cholesterol (mg/dL) ^†^	62.0	±11.2	56.2	±12.1 *^a^*	41.3	±7.09 *^c^*	44.9	±9.60 *^d^*	<0.001
LDL cholesterol (mg/dL)	112.6	±37.3	126.8	±34.0	115.5	±29.8	139.7	±32.0 *^d^*	0.016
Glucose (mg/dL) ^†^	86.0	±6.51	97.0	±6.95 *^a^*	87.0	±11.4	104.6	±11.3 *^bd^*	<0.001
Insulin (μIU/dL) ^†^	10.6	±3.23	12.1	±4.62	11.5	±2.61	13.7	±5.72	0.086
HOMA-IR ^†^	2.24	±0.71	2.92	±1.21 *^a^*	2.51	±0.88	3.52	±1.50 *^d^*	<0.001
C-peptide (ng/mL) ^†^	1.63	±0.45	1.94	±0.66 *^a^*	1.70	±0.30	2.31	±0.69 *^d^*	0.002
AST (IU/L) ^†^	20.5	±3.92	20.2	±5.58	22.3	±5.77	23.3	±9.69 *^d^*	0.317
ALT (IU/L) ^†^	17.4	±7.54	16.8	±7.14	23.0	±11.3	22.3	±13.9 *^d^*	0.055
γGTP (U/L) ^†^	21.1	±14.8	19.2	±14.0	20.0	±7.00	25.7	±14.6	0.002
Adiponectin (ng/mL) ^†^	11.8	±9.77	7.92	±4.64 *^a^*	5.79	±4.35	6.74	±5.21 *^d^*	0.005
Leptin (ng/mL) ^†^	18.1	±6.31	22.5	±10.8	17.6	±1.48	24.1	±8.62	0.105
8-epi-PGF_2α_ (pg/mg creatinine) ^†^	1403.9	±545.5	1510.0	±487.1	1128.1	±74.7	1675.4	±535.1 *^b^*	0.039
MDA (nmol/mL) ^†^	6.81	±1.47	7.72	±2.68 *^a^*	6.33	±0.58	8.79	±1.95 *^bd^*	0.001
Ox-LDL (U/L) ^†^	58.8	±19.0	65.4	±20.3	61.7	±18.1	78.7	±21.7 *^d^*	0.003
Measurements from DEXA and CT									
Fat percentage (%) ^†^	33.4	±3.23	33.1	±3.13	33.3	±0.51	33.5	±3.04	0.992
Fat mass (g)	22,968.0	±3474.9	22,769.7	±3327.5	22,274.3	±2733.5	23,457.7	±3207.0	0.759
Lean body mass (g) ^†^	43,540.4	±4010.1	43,641.5	±4032.6	42,553.3	±4341.4	44,347.4	±4358.8	0.767
L1 vertebra									
Whole fat area (cm^2^)	237.3	±57.9	237.8	±59.9	279.7	±79.1	260.6	±38.7 *^d^*	0.070
Visceral fat area (cm^2^)	93.9	±45.2	91.6	±34.8	135.4	±60.5	100.7	±29.1	0.232
Subcutaneous fat area (cm^2^)	143.4	±26.8	146.2	±36.7	144.3	±22.8	160.0	±30.4 *^d^*	0.130
L4 vertebra									
Whole fat area (cm^2^)	300.9	±46.1	323.4	±49.5	329.9	±48.9	344.7	±34.5 *^d^*	0.010
Visceral fat area (cm^2^)	78.3	±26.6	94.0	±27.1 *^a^*	131.6	±14.3 *^c^*	107.9	±24.7 *^d^*	<0.001
Subcutaneous fat area (cm^2^)	222.6	±42.2	229.4	±43.2	198.3	±34.7	236.8	±32.1	0.270

Mean ± S.D. ^†^ tested following logarithmic transformation. *p*-values derived from Kruskal–Wallis ANOVA among the four groups. *^a^ p* < 0.05 between the metabolically healthy obesity (MHO) groups according to the Mann–Whitney *U*-test. *^b^ p* < 0.05 between the metabolically unhealthy obesity (MUO) groups according to the Mann–Whitney *U*-test. *^c^ p* < 0.05 between the GC groups according to the Mann–Whitney *U*-test. *^d^ p* < 0.05 between the GG groups according to the independent *t*-test.
